# Inter-individual variability of neurotransmitter receptor and transporter density in the human brain

**DOI:** 10.1007/s00429-025-03069-2

**Published:** 2026-01-13

**Authors:** Justine Y. Hansen, Jouni Tuisku, Jarkko Johansson, Zeyu Chang, Colm J. McGinnity, Vincent Beliveau, Synthia Guimond, Melanie Ganz, Martin Nørgaard, Marian Galovic, Gleb Bezgin, Sylvia M. L. Cox, Jarmo Hietala, Marco Leyton, Eliane Kobayashi, Pedro Rosa-Neto, Thomas Funck, Nicola Palomero-Gallagher, Gitte M. Knudsen, Paul Marsden, Alexander Hammers, Lauri Nummenmaa, Lauri Tuominen, Bratislav Misic

**Affiliations:** 1 Montréal Neurological Institute, McGill University, Montréal, QC Canada; 2 Turku University Hospital, University of Turku, Turku, Finland; 3 Umeå University, Umeå, Sweden; 4 King’s College London and Guy’s and St Thomas’ PET Centre, London, UK; 5 Medical University of Innsbruck, Innsbruck, Austria; 6 Neurobiology Research Unit, Copenhagen University Hospital Rigshospitalet, Copenhagen, Denmark; 7 Department of Psychiatry, The Royal’s Institute of Mental Health Research, University of Ottawa, Ottawa, ON Canada; 8 Department of Psychoeducation and Psychology, University of Quebec in Outaouais, Gatineau, QC Canada; 9 Department of Computer Science, University of Copenhagen, Copenhagen, Denmark; 10 Molecular Imaging Branch, National Institute of Mental Health (NIMH), Rockville, USA; 11 Clinical Neuroscience Center, University Hospital Zurich, Zurich, Switzerland; 12 UCL Queen Square Institute of Neurology, London, UK; 13 Department of Psychiatry, McGill University, Montréal, QC Canada; 14 Department of Neurology and Peter O’Donnell Jr. Brain Institute, University of Texas Southwestern Medical Center, Dallas, USA; 15 McGill University Research Centre for Studies in Aging, Alzheimer’s Disease Research Unit, Douglas Research Institute, Le Centre Intégré Universitaire de Santé et de Services Sociaux (CIUSSS) de l’Ouest-de-l’île-de-Montréal, Montreal, USA; 16 Center for the Developing Brain, Child Mind Institute, New York, USA; 17 Institute of Neuroscience and Medicine (INM-1), Research Centre Jülich, Jülich, Germany; 18 C. and O. Vogt Institute for Brain Research, Medical Faculty, University Hospital Düsseldorf, Heinrich-Heine University Düsseldorf, Düsseldorf, Germany; 19 Institute of Clinical Medicine, University of Copenhagen, Copenhagen, Denmark; 20 Research Department of Biomedical Computing and Research Department of Early Life Imaging, King’s College London, School of Biomedical Engineering and Imaging Sciences, London, UK

**Keywords:** Neurotransmitter receptors, Inter-individual variation, Spatial variation, PET imaging, Brain mapping

## Abstract

Neurotransmitter receptors guide the propagation of signals between brain regions. Mapping receptor distributions in the brain is therefore necessary for understanding how neurotransmitter systems mediate the link between brain structure and function. Normative receptor density can be estimated using group averages from Positron Emission Tomography (PET) imaging. However, the generalizability and reliability of group-average receptor maps depends on the inter-individual variability of receptor density, which is currently unknown. Here we collect group standard deviation brain maps of PET-estimated protein abundance for 12 different neurotransmitter receptors and transporters across 7 neurotransmitter systems, including dopamine, serotonin, acetylcholine, glutamate, GABA, cannabinoid, and opioid. We illustrate how cortical and subcortical inter-individual variability of receptor and transporter density varies across brain regions and across neurotransmitter systems. We complement inter-individual variability with inter-regional variability, and show that receptors that vary more across brain regions than across individuals also demonstrate greater out-of-sample spatial consistency. Altogether, this work quantifies how receptor systems vary in healthy individuals, and provides a means of assessing the generalizability of PET-derived receptor density quantification.

**Supplementary Information:**

The online version contains supplementary material available at 10.1007/s00429-025-03069-2.

## Introduction

Neurotransmitter receptors modulate neuronal activity, guide synaptic wiring, and mediate brain-wide communication. Mapping neurotransmitter receptor distributions in the brain is therefore necessary for understanding how chemoarchitecture shapes brain structure and function. We recently collated a Positron Emission Tomography (PET) atlas of in vivo whole-brain neurotransmitter receptor and transporter densities across 19 unique receptors and transporters and 9 neurotransmitter systems (Hansen et al. 2022a; Markello et al. 2022). This atlas is widely used for studying chemoarchitectonic mechanisms underlying, for example, neural rhythms (Shafiei et al. 2023), pharmacological perturbations (Tuominen et al. 2025; Luppi et al. 2023), energy metabolism (Castrillon et al. 2023), cognition (Yang et al. 2023), and multiple diseases and disorders (Ricard et al. 2024; Hansen et al. 2022; Morys et al. 2024; Jiang et al. 2024; Wiesman et al. 2024).

Nevertheless, brain anatomy and function vary across individuals, manifesting as individual differences in cognition and behaviour (Mueller et al. 2013; Bethlehem et al. 2022; Segal et al. 2025). In addition, brain regions and systems develop at different rates, and are differentially subjected to influence from the environment (e.g. via sensory stimuli) and transcriptomic programs (Buckner and Krienen 2013; Sydnor et al. 2021). Inter-individual variability in receptor density may therefore be greater in some brain regions than in others. Some inferences on the inter-individual variability of receptor density can be made from group-average receptor density maps alone: group receptor density brain maps can be compared across sites, PET tracers, imaging modalities, and even across biological features (e.g. receptor density versus protein-coding gene expression) (Hansen et al. 2022a, b; Murgaš et al. 2022; Beliveau et al. 2017; Nørgaard et al. 2021). However, these strategies can only assess the spatial similarity of brain maps rather than the inter-individual variability of regional receptor density.

To better understand how receptor abundance varies across individuals, we collate group standard deviation maps for 12 neurotransmitter receptors and transporters across 7 neurotransmitter systems and nearly 700 individuals. We show cortical and subcortical brain maps of inter-individual receptor abundance variability, and benchmark receptor variability across PET tracers. We then compare inter-individual and inter-regional variability. By interpreting the present findings alongside previous work comparing spatial distributions of receptors, we provide receptor-specific hypotheses for sources of variability. Altogether, this work serves as a reference point for assessing receptor and transporter measurement generalizability in the human brain.

**Fig. 1:**
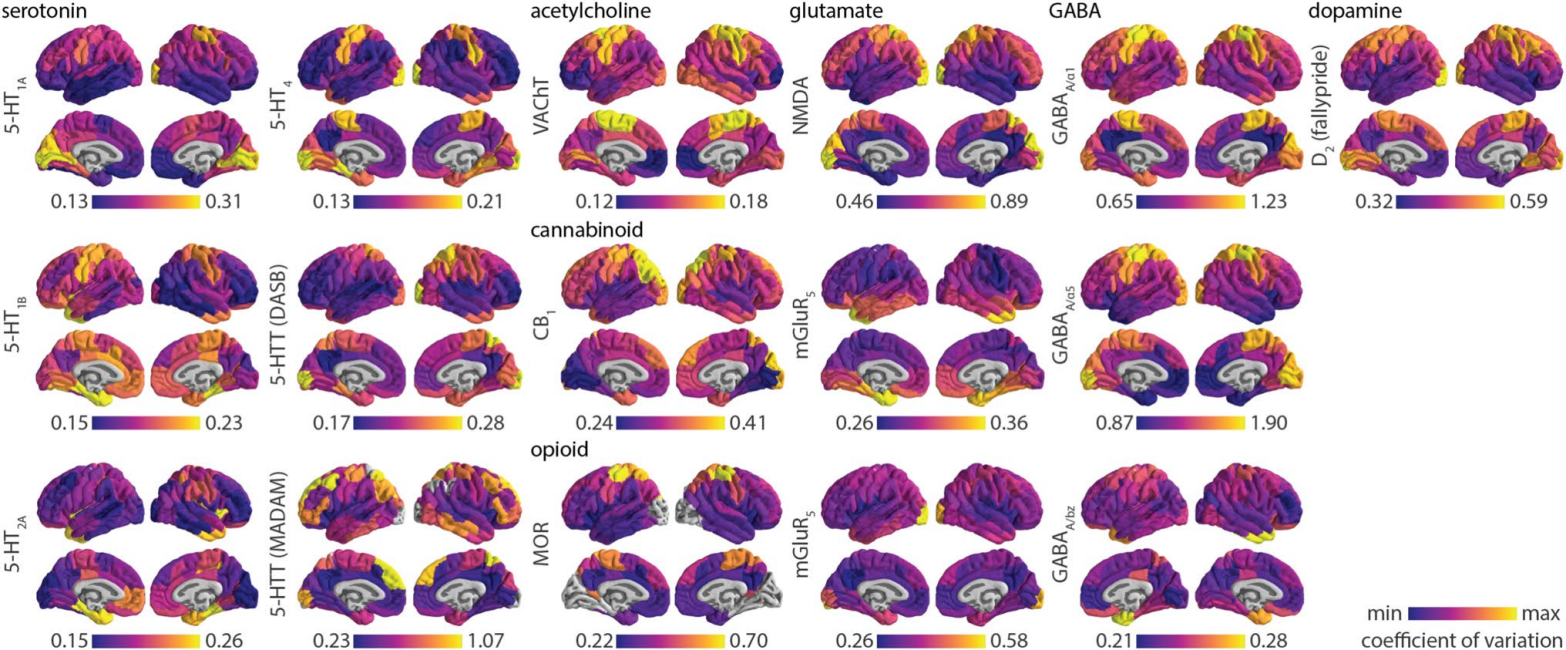
Inter-individual coefficient of variation of receptor/transporter density in the cortex. Inter-individual coefficient of variation is defined as the population standard deviation of tracer binding normalized by population mean, and is calculated for every cortical region. Each coefficient of variation brain map is min-max scaled to showcase the spatial organization of inter-individual variability of neurotransmitter systems. Grey colours reflect regions that have been omitted due to either unstable coefficient of variation or tracer binding quantification reference regions (see *Methods* for details). Two tracers that map 5-HTT were included; tracer names are written in parentheses. GABA$_\text {A}$ receptors were mapped according to two different subunits ($\alpha _{1}$ and $\alpha _{5}$) as well as the benzodiazepine binding site (bz). D$_{2}$ [$^{11}$C]raclopride tracer data is not shown due to high non-displaceable binding in the cortex

## Results

**Fig. 2:**
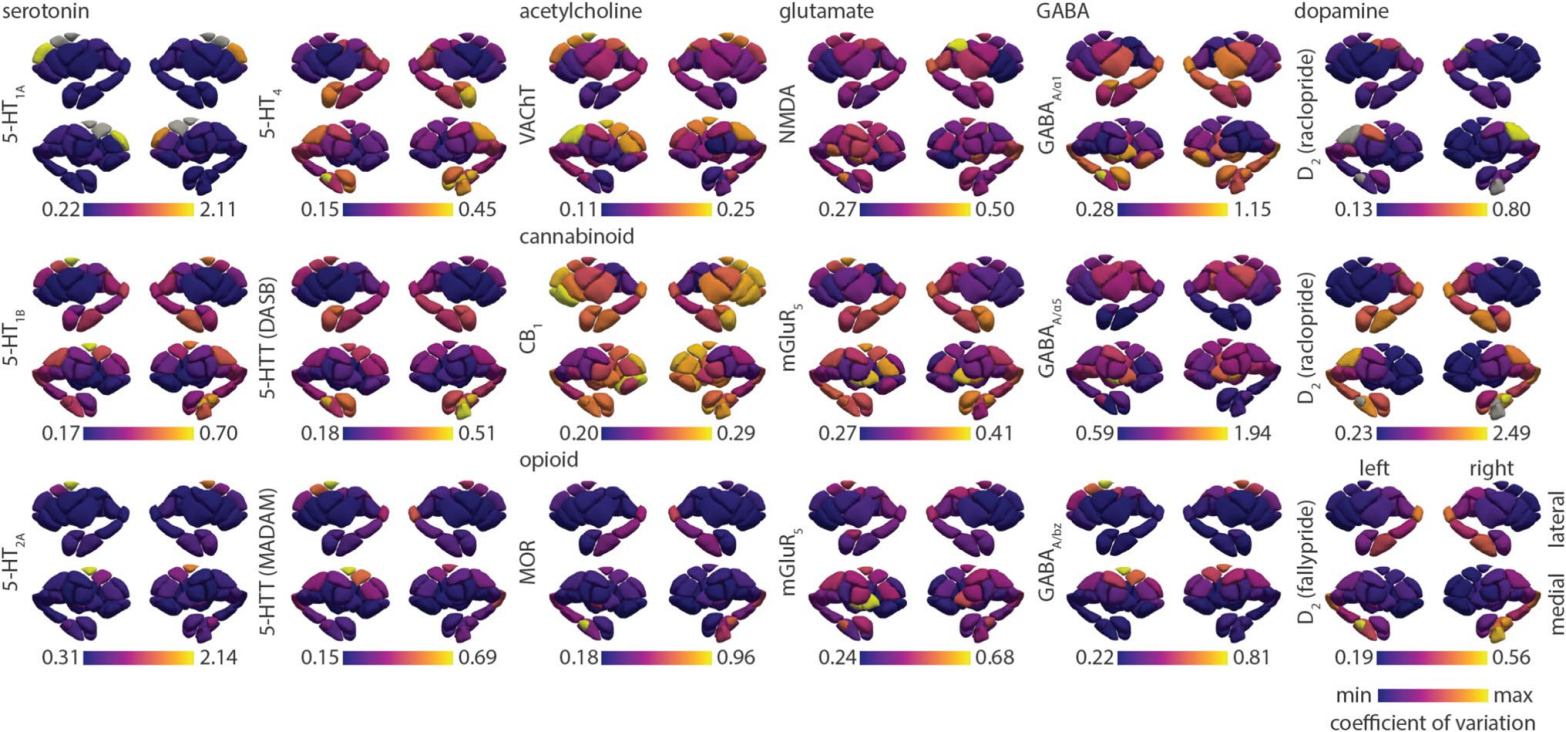
Inter-individual coefficient of variation of receptor/transporter density in the subcortex. Inter-individual coefficient of variation is defined as the population standard deviation of tracer binding normalized by population mean, and is calculated for every subcortical region. Each coefficient of variation brain map is min-max scaled to showcase the spatial organization of inter-individual variability of neurotransmitter systems. Grey colours reflect regions that have been omitted due to unstable coefficient of variation (see *Methods* for details). Tracer names are included in parentheses for 5-HTT and D$_{2}$. GABA$_\text {A}$ receptors were mapped according to two different subunits ($\alpha _{1}$ and $\alpha _{5}$) as well as the benzodiazepine binding site (bz). Note that D$_{2}$ [$^{11}$C]raclopride tracer is only sensitive within the striatum

**Fig. 3:**
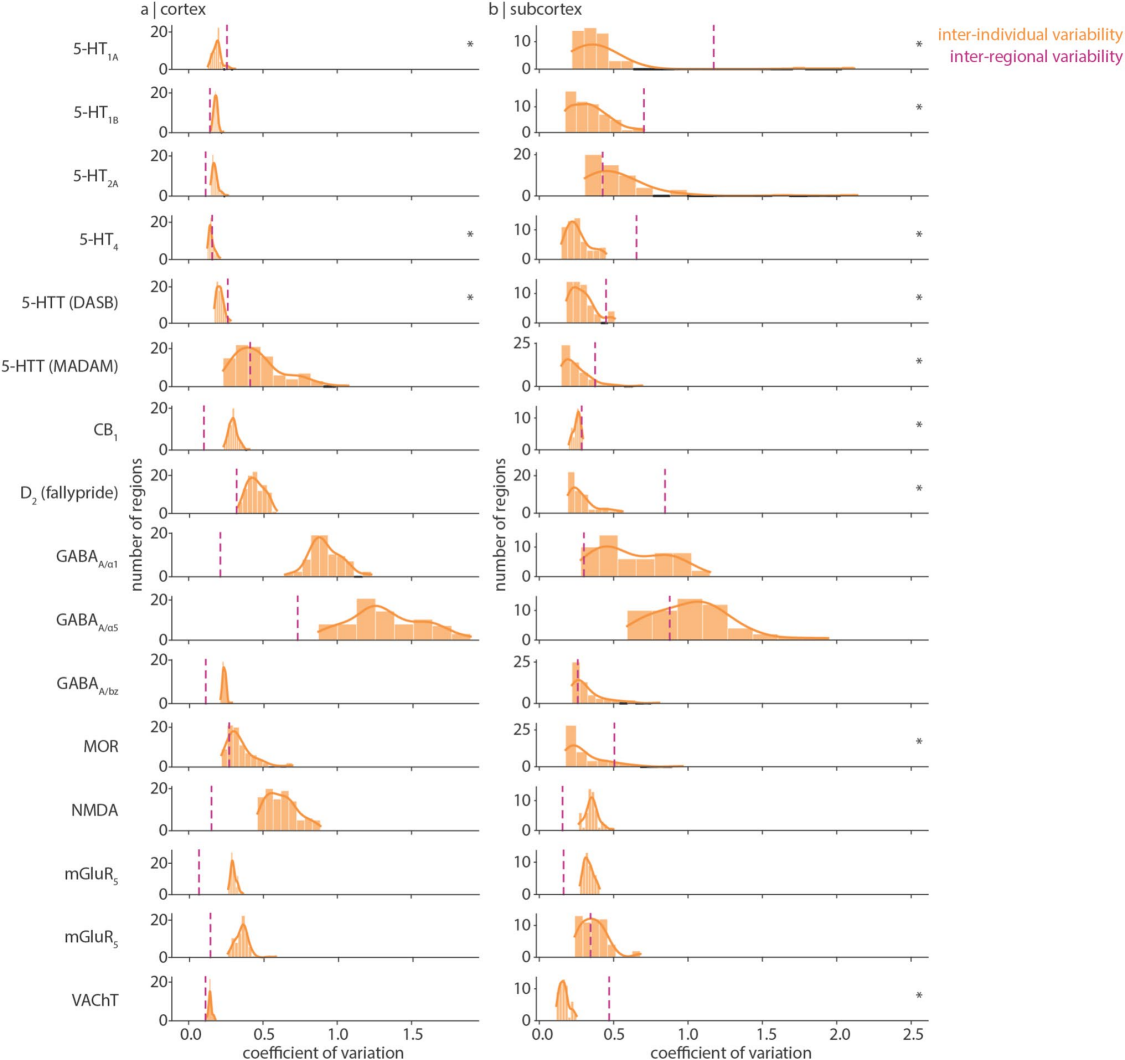
Distributions of inter-individual coefficient of variation. For each receptor and transporter (rows), the distribution of within-region inter-individual coefficient of variation is shown in orange for (a) cortical regions and (b) subcortical regions. These are the same data as shown in Fig. 1 and Fig. 2. A kernel density is estimated for each distribution (solid orange line). The y-axis represents the number of brain regions within each histogram bin, and the smooth curve represents the probability density estimate of the underlying histogram. The dashed purple line represents the inter-regional coefficient of variation. Asterisks indicate receptors/transporters whose inter-regional coefficient of variation is significantly greater than a null distribution of mean bootstrapped inter-individual coefficient of variation

**Fig. 4:**
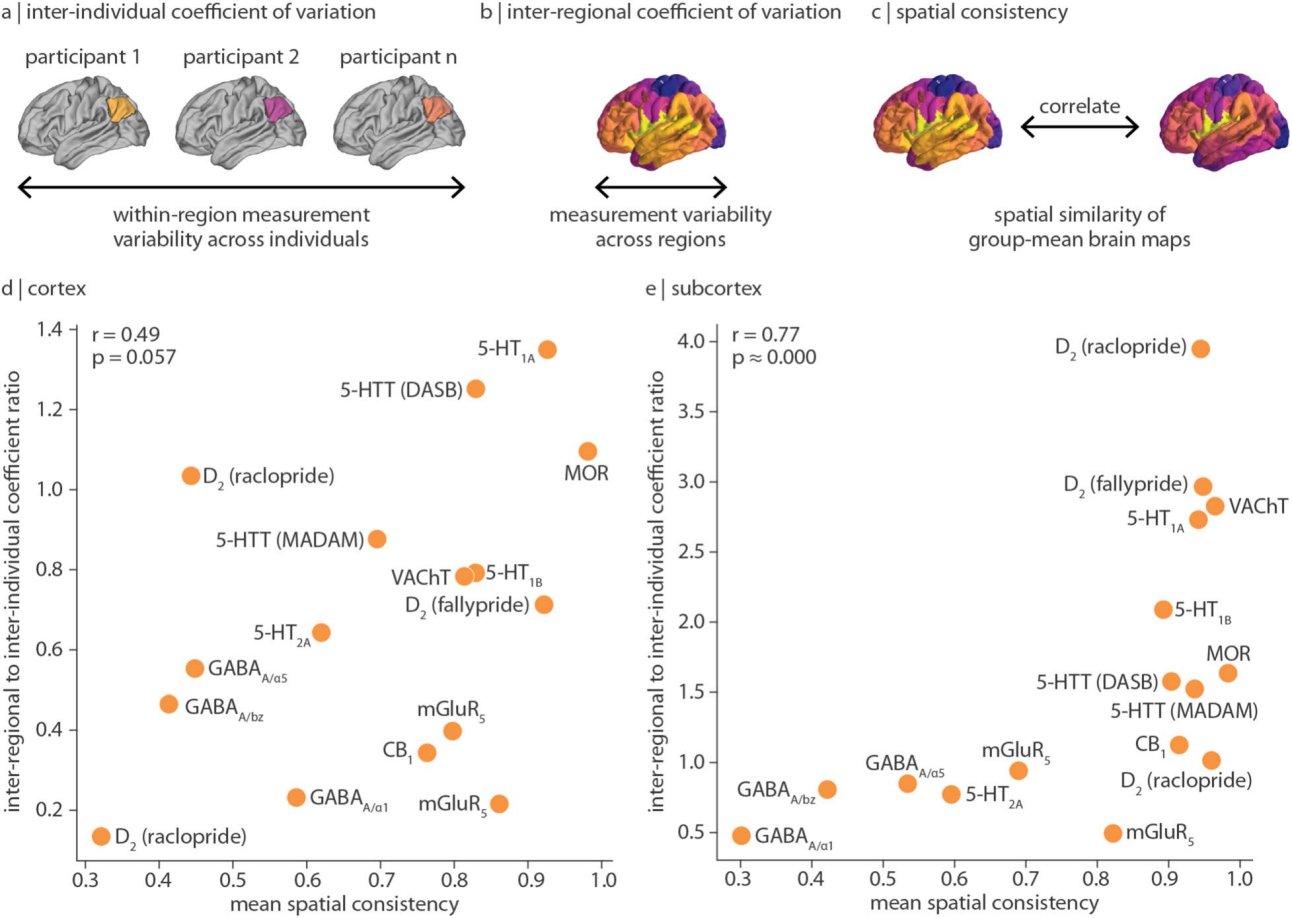
Comparing inter-regional and inter-individual variation of receptor/transporter density | A schematic illustrating three perspectives of variability: (**a**) inter-individual coefficient of variation quantifies within-region measurement variability across participants; (**b**) inter-regional coefficient of variation quantifies variability of group-averaged measurements across brain regions; and (**c**) spatial consistency quantifies the similarity of group-averaged measurements of the same receptor/transporter. For (**d**) cortex and (**e**) subcortex, regional-to-population coefficient of variation ratio (*y*-axis) is defined as the inter-regional coefficient of variation (dashed purple line in Fig. 3) normalized by the mean inter-individual coefficient of variation (mean of orange bars in Fig. 3). Values above 1 represent receptors/transporters that vary more across regions than across individuals, and vice versa for values below 1. Note that *y*-axis limits are different in panels (**d**) and (**e**). Next, mean spatial consistency is defined as the mean pairwise spatial Spearman’s correlation of group-average tracer images of the same receptor/transporter (*x*-axis). Tracers used for each out-of-sample comparison are detailed in Table S1. Note that GABA$_\text {A}$ images map different subunits of the GABA$_\text {A}$ receptor—these receptor subtypes demonstrate unique expression profiles, resulting in lower spatial consistency (Sieghart and Sperk 2002)

We collated group standard deviation maps of PET-derived neurotransmitter receptor and transporter densities from a total of 12 different receptors/transporters across 7 neurotransmitter systems, including dopamine, serotonin, acetylcholine, glutamate, GABA, cannabinoid, and opioid (Table 1). All mean and standard deviation maps are parcellated according to 100 cortical regions (Schaefer et al. 2018) and 54 subcortical regions (Tian et al. 2020) (note that allocortex (e.g. hippocampus) is included in the subcortical atlas). Given that standard deviations scale with the mean (Fig. S1,S2), we normalize standard deviation by the mean, resulting in a brain map of the within-region inter-individual coefficient of variation for each neurotransmitter receptor and transporter (Fig. 1, Fig. 2). In both cortex and subcortex, inter-individual coefficient of variation is heterogeneously distributed and highly organized across brain regions. For many receptors and transporters, cortical coefficient of variation appear greatest in unimodal brain regions, including primary somatomotor and somatosensory cortex as well as primary visual cortex (Fig. 1). Meanwhile, subcortical coefficient of variation is often greatest in ventral structures as well as the caudate (Fig. 2).

In Fig. 3 we show the distribution of cortical and subcortical coefficients of variation for each neurotransmitter receptor and transporter. Density measurements in subcortical structures often vary more than in cortical structures. Within the cortex, inter-individual coefficient of variation is generally low (around 0.2), with some receptors/transporters showing moderate variation (around 0.4, e.g. MOR, CB$_{1}$), and some high variation ($>0.5$, e.g. NMDA, GABA$_\text {A}$ $\alpha _{1}$ and $\alpha _{5}$ subunits). We confirm that the D$_{2}$ tracer [$^{11}$C]raclopride, which is only suitable for quantification of striatal D$_{2}$ receptors (Dagher and Palomero-Gallagher 2020), shows greatest variation outside of the striatum, as a result of increased measurement noise (Fig.S3). In addition, we find that different tracers that bind to the same protein can show different amounts of inter-individual variability, possibly due to differences in study design and preprocessing (e.g. 5-HTT [$^{11}$C]MADAM tracer binding is more variable than 5-HTT [$^{11}$C]DASB tracer binding within the cortex (Nørgaard et al. 2019, 2020)).

Inter-individual variance of a regional measurement is better interpreted in light of the receptor/transporter’s measurement variability across brain regions. To develop this point further, consider a group-averaged measurement with low variation across brain regions (i.e. is approximately homogeneously expressed in the brain) but high variation across individuals. This measurement will have a highly variable spatial profile (i.e. brain map) from one individual to the next. On the other hand, if a measurement varies more across regions than individuals, the regional rank order of protein density will remain similar in all individuals; that is, this measurement will be consistently spatially expressed across individuals. To quantify receptor/transporter density variability across regions, we calculate inter-regional coefficient of variation: the standard deviation of group-averaged receptor/transporter density across brain regions normalized by the mean (Fig. 3 dashed vertical lines; see also schematics in Fig. 4a–c). We find that, in the cortex, many receptors/transporters show similar or greater variability across individuals than regions; indeed, only 3/16 receptors/transporters demonstrate significantly greater inter-regional coefficient of variation than inter-individual coefficient of variation. Within the subcortex however, receptor/transporter density often varies less across individuals than across regions (9/16 receptors/transporters demonstrate significantly greater inter-regional coefficient of variation than inter-individual coefficient of variation). This suggests that, although population variance is generally greater in subcortex than in cortex (Fig. 3 yellow bars), subcortical receptor/transporter expression is likely to be stably spatially expressed. Indeed, we find that the ratio of spatial variation to population variation is positively correlated with the out-of-sample consistency of a receptor/transporter’s spatial distribution (i.e. mean pairwise Spearman correlation of receptor/transporter brain maps from different cohorts. $r=0.49$, $p=0.057$ within cortex; $r=0.77$, $p\approx 0$ within subcortex; Fig. 4). Note the non-significant relationship in the cortex, which may be due to lower sensitivity and reliability of certain tracers (e.g. [$^{11}$C]raclopride), but also highlights exceptions such as glutamatergic mGluR$_{5}$ and endocannabinoid CB$_{1}$, both of which demonstrate highly replicable spatial patterns but low regional-to-population coefficient of variation ratio.

## Discussion

In the present report, we estimate standard deviation maps for 12 unique neurotransmitter receptors and transporters to better understand how receptor and transporter density varies across individuals. We show that receptor and transporter variability is heterogeneous across brain regions and systems. Cortical receptor/transporter density typically varies more across individuals than across brain regions, while subcortical receptor/transporter density typically varies less across individuals than across regions. Finally, we show that receptors/transporters that vary more across regions than individuals are also more consistently spatially mapped.

The recent proliferation of group-averaged “reference” brain maps make it possible to spatially relate diverse brain phenotypes with one another (Markello et al. 2022; Hansen et al. 2022a; Hansen and Misic 2025). However, the interpretation of such associations is dependent on the generalizability and reliability of these reference maps, which are rarely accompanied by estimates of inter-individual variability (Segal et al. 2025). Here we aim to rectify this limitation by retroactively compiling standard deviation maps for previously shared mean receptor density brain maps (see Hansen et al. (2022a)). We find that inter-individual variability of regional receptor density is organized along specific anatomical landmarks, such that some brain areas vary more across people than others. While inter-individual variability of structural and functional cortical features is generally greater in transmodal cortex and lower in unimodal cortex (Cui et al. 2020; Mueller et al. 2013; Reardon et al. 2018; Karahan et al. 2022; Huang et al. 2025), we find that the opposite is true for many neurotransmitter receptors and transporters (Fig. 1). This difference in findings may in part be due to the mathematical relationship between coefficient of variation and standard deviation. For multiple receptors where unimodal cortical regions have large coefficient of variation (e.g. 5-HT$_\text {1A}$, 5-HT$_\text {1B}$, 5-HT$_{4}$, MOR, NMDA, GABA$_\text {A}$, D$_2$), these same regions have low standard deviation (Fig.S1). However, given the fact that standard deviation scales with the mean (Eisler et al. 2008), a mean-normalized measurement such as the coefficient of variation is more interpretable than the standard deviation. How structural and functional connectivity varies with respect to mean connectivity remains unknown. Inter-individual variability of receptor expression may also be larger in unimodal than transmodal cortex because receptor expression is tightly coupled to sensory input (Peckol et al. 2001; Tyler et al. 2007). Individual differences in environmental and external stimuli may therefore exert a greater influence on receptor expression in unimodal over transmodal cortex. As brain maps of inter-individual variability are generated and shared (Karahan et al. 2022; Sydnor et al. 2021; Monaghan et al. 2024), we will better understand how variability varies across brain regions and biological systems.

By combining evidence from multiple lines of analysis, we are able to generate hypotheses regarding the source of variability (e.g. measurement or biological) of different receptors’ expression. Aside from true biological variability, measurements of inter-individual variability may be influenced by sample size or age (although in this dataset we do not find statistically significant relationships for either (Fig.S4)), tracer kinetics (e.g. [$^{11}$C]MADAM versus [$^{11}$C]DASB when measuring 5-HTT density (Nørgaard et al. 2019, 2020)), scanner, and PET processing pipeline (including e.g. template space and registration method). We can therefore aid our interpretation of variability sources with reported findings that test out-of-sample spatial replicability using other measurements techniques (e.g. autoradiography, as shown in (Hansen et al. 2022b; Nørgaard et al. 2021; Beliveau et al. 2017)) and proxies of receptor abundance (e.g. gene expression, as shown in (Rizzo et al. 2014; Hansen et al. 2022b; Murgaš et al. 2022)). Take for example serotonergic 5-HT$_\text {1A}$ density: this receptor is stably expressed across both brain regions and individuals (coefficient of variation around 0.2), spatially replicable across both PET ($r>0.9$) and autoradiography ($r>0.6$) cohorts, and strongly correlated with its protein-coding gene ($r=0.88$), indicating a protein with approximately the same regional receptor abundance in any brain (i.e. low biological variability, low measurement variability, and conserved spatial expression) (Hansen et al. 2022a, b; Murgaš et al. 2022; Beliveau et al. 2017). Similarly, the endocannabinoid receptor CB$_{1}$, glutamatergic receptor mGluR$_{5}$, and opioid receptor MOR demonstrate spatial consistency (mean $r>0.75$) and CB$_{1}$ and MOR also demonstrate high coexpression with their protein-coding genes (*CNR1* ($r=0.74$) and *OPRM1* ($r=0.84$) respectively, as reported in Hansen et al. (2022b)). However, their regional receptor abundance is variable across people (coefficient of variation around 0.4). This suggests that, while the spatial distributions of these proteins are consistent, they may exhibit an individual-specific baseline shift (i.e. high biological variability, low measurement variability, and conserved spatial expression). This is supported also by the fact that mGluR$_{5}$ and CB$_{1}$ appear as outliers in the correlation between mean spatial consistency and inter-regional to inter-individual coefficient of variation ratio (Fig. 4d). Finally, there are receptors that are systematically inconsistently expressed. Ionotropic (and heteromeric) receptors GABA$_\text {A}$ ($\alpha _{1}$ and $\alpha _{5}$ subunits) and NMDA show high population variability in regional receptor abundance (coefficient of variation $>0.5$) and GABA$_\text {A}$’s spatial patterning is only moderately replicable in separate PET ($r\approx 0.5$) and autoradiography ($r=0.20$) cohorts. Such inconsistent measurements may reflect noise (Schoenberger et al. 2018), individual-specific expression (Arumuham et al. 2023; Mosconi et al. 2024; Kaasinen et al. 2021), protein turnover rate (i.e. temporal variability), or individual differences in receptor subunit composition.

We end with a note on interpretation. First, while we show brain maps of inter-individual coefficient of variation in the cortex and subcortex (Figs. 1, 2), these maps are min-max scaled and in many cases (e.g. the serotonergic receptors), the inter-individual coefficient of variation is consistently very low. Figure 3 should be used to compare the variability across tracers. Second, our measurement of inter-individual variability is agnostic to whether the source of variability is individual differences, measurement noise, or study design (e.g. modelling technique) (Nørgaard et al. 2020). To better assess the generalizability and replicability of receptor brain maps, we apply our own out-of-sample comparisons and we draw on our earlier work comparing alternative PET tracers, imaging modalities, and protein-coding gene expression (Hansen et al. 2022a, b). Third, due to ethical restrictions in sharing individual data, we are unable to test whether receptor binding is normally distributed across individuals. Individual outliers may therefore skew the standard deviation.

In summary, we assemble an atlas of neurotransmitter receptor and transporter density variability. This atlas complements our previously published atlas of whole-brain receptor/transporter densities (Hansen et al. 2022a). Our work sheds light on how receptor systems vary in healthy individuals, and provides a means of assessing the generalizability of PET-derived receptor density quantification.

**Table 1:** Neurotransmitter receptors and transporters included in analyses | BP$_\text {ND} =$ non-displaceable binding potential; V$_\text {T} =$ tracer distribution volume; B$_\text {max} =$ density (pmol/ml) converted from binding potential using autoradiography-derived densities; SUVR $=$ standard uptake value ratio

Receptor transporter	Neurotransmitter	Tracer	Measure	*N*	Age	References
5-HT$_\text {1A}$	Serotonin	[$^{11}$C]CUMI-101	B$_\text {max}$	8 (5)	$28.4 \pm 8.8$	Beliveau et al. (2017)
5-HT$_\text {1B}$	Serotonin	[$^{11}$C]AZ10419369	B$_\text {max}$	36 (12)	$27.8 \pm 6.9$	Beliveau et al. (2017)
5-HT$_\text {2A}$	Serotonin	[$^{11}$C]Cimbi-36	B$_\text {max}$	29 (14)	$22.6 \pm 2.7$	Beliveau et al. (2017)
5-HT$_{4}$	Serotonin	[$^{11}$C]SB207145	B$_\text {max}$	59 (18)	$25.9 \pm 5.3$	Beliveau et al. (2017)
5-HTT$^*$	Serotonin	[$^{11}$C]DASB	B$_\text {max}$	100 (71)	$25.1 \pm 5.8$	Beliveau et al. (2017)
5-HTT$^*$	Serotonin	[$^{11}$C]MADAM	BP$_\text {ND}$	49 (24)	$39.3 \pm 6.4$	Majuri et al. (2017); Tuominen et al. (2014)
CB$_1$	Cannabinoid	[$^{18}$F]FMPEP-d$_{2}$	V$_\text {T}$	20 (0)	$24.4 \pm 3.0$	Pekkarinen et al. (2023)
D$_{2}$	Dopamine	[$^{11}$C]raclopride	BP$_\text {ND}$	16 (7)	$32.7 \pm 8.8$	Hamati et al. (2025)
D$_{2}$	Dopamine	[$^{11}$C]raclopride	BP$_\text {ND}$	47 (0)	$23.5 \pm 2.5$	Alakurtti et al. (2011); Bäckman et al. (2017, 2011)
D$_{2}$	Dopamine	[$^{18}$F]fallypride	BP$_\text {ND}$	49 (33)	$18.4 \pm 0.6$	Jaworska et al. (2020)
GABA$_\text {A}/ \alpha _{1}$	GABA	[$^{11}$C]Ro15 4513	V$_\text {T}$	27 (1)	$45.96 \pm 7.4$	McGinnity et al. (2021); Chang et al. (2025)
GABA$_\text {A}/\alpha _{5}$	GABA	[$^{11}$C]Ro15 4513	V$_\text {T}$	27 (1)	$45.96 \pm 7.4$	McGinnity et al. (2021); Chang et al. (2025)
GABA$_\text {A/BZ}$	GABA	[$^{11}$C]flumazenil	B$_\text {max}$	16 (9)	$26.6 \pm 8$	Nørgaard et al. (2021)
NMDA	Glutamate	[$^{18}$F]GE-179	V$_\text {T}$	29 (8)	$40.9 \pm 12.7$	Galovic et al. (2021a, 2021b); McGinnity et al. (2014)
mGluR$_{5}$	Glutamate	[$^{11}$C]ABP688	BP$_\text {ND}$	27 (12)	$54.6 \pm 13.4$	DuBois et al. (2016)
mGluR$_{5}$	Glutamate	[$^{11}$C]ABP688	BP$_\text {ND}$	73 (48)	$19.9 \pm 3.0$	Smart et al. (2019)
MOR	Opioid	[$^{11}$C]carfentanil	BP$_\text {ND}$	86 (42)	$35.6 \pm 9.9$	Tuominen et al. (2014); Majuri et al. (2017); Johansson et al. (2019); Lamusuo et al. (2017)
VAChT$^{*}$	Acetylcholine	[$^{18}$F]FEOBV	SUVR	25 (8)	$36.6 \pm 9.7$	Saint-Georges et al. (2025)

Values in parentheses (under *N*) indicate number of females. Asterisks indicate transporters

## Methods

### PET data acquisition

Our group had previously assembled group-averaged PET tracer images for 19 neurotransmitter receptors and transporters from research groups and PET imaging centers globally (Hansen et al. 2022a). In an effort to better understand how these measurements vary across individuals, we recontacted all collaborators who had contributed mean receptor maps and asked whether they would be interested in providing group mean and standard deviation images for each tracer. Altogether we compiled 18 tracer mean and standard deviation images, encompassing 12 unique neurotransmitter receptors and transporters, and 7 neurotransmitter systems. Each study, the associated receptor/transporter, tracer, number of healthy participants, age, and reference with full methodological details of data acquisition can be found in Table 1. In all cases, only scans from healthy participants were included. Group mean and standard deviation images were registered to MNI152NLin6Asym space, then parcellated according to 100 cortical regions as defined by the Schaefer parcellation (Schaefer et al. 2018) and 54 subcortical regions as defined by the Melbourne Subcortex Atlas S4 (Tian et al. 2020).

We note some tracer-specific special cases: (1) while tracer binding for most neurotransmitter receptors is estimated using the cerebellum as the reference region, the mu-opioid receptor (MOR) is measured using the occipital cortex as the reference region. We therefore set all regions in the occipital cortex to NaN. (2) Three dopaminergic D$_{2}$ images were shared, two measured with the tracer [$^{11}$C]raclopride and one measured with the tracer [$^{18}$F]fallypride. Due to the lower affinity of [$^{11}$C]raclopride to D$_{2}$ receptors, this tracer can only reliably estimate binding in regions with high D$_{2}$ density (i.e. the striatum) (Palomero-Gallagher and Zilles 2019). [$^{11}$C]raclopride measurements outside of the striatum are therefore expected to demonstrate large variation across participants. On the other hand, [$^{18}$F]fallypride is primarily suitable for estimation of extra-striatal D$_{2}$ receptors (Jaworska et al. 2020; Vernaleken et al. 2011). (3) Two serotonergic 5-HTT images acquired using different tracers ([$^{11}$C]DASB and [$^{11}$C]MADAM) were shared. We include both for comparison. (4) Two subunits ($\alpha _{1}$ and $\alpha _{5}$) of the GABA$_\text {A}$ receptor were mapped using a single PET tracer [$^{11}$C]Ro15-4513 by way of spectral analysis (McGinnity et al. 2021); we include both for comparison. We also include [$^{11}$C]flumazenil, a tracer that binds to the benzodiazepine (bz) binding site of GABA$_\text {A}$ receptors (Nørgaard et al. 2021). Although subunits $\alpha _{1}$, $\alpha _{5}$, and benzodiazapine are all part of the GABA$_\text {A}$ receptor, they demonstrate diverse spatial profiles (Sieghart and Sperk 2002). (5) Two mGluR$_{5}$ images were shared, both measured using [$^{11}$C]ABP688; we include both for comparison.

### Spatial consistency

In Fig. 4, we compare inter-regional to inter-individual coefficient of variation ratio to the mean spatial consistency of a tracer image. Spatial consistency refers to the spatial correlation between group-mean brain maps. For the set of images with both group mean and group standard deviation (i.e. every point in Fig. 4d–e; “Original map” in Table S1), we collate a sample of group mean images of the same receptor/transporter (“Other maps(s)” in Table S1). The sample of “Other maps” is created from a combination of alternative group mean images used in the present manuscript (when duplicates exist) and group mean images shared in Hansen et al. (2022a) but not used in the present work.

Mean spatial consistency is defined as the average spatial correlation (Spearman’s *r*) between the original map and all maps listed under “Other map(s)”. (Note that in the case of 5-HT$_\text {1A}$, there is only one alternative map, therefore mean spatial consistency is simply the spearman correlation between two group-mean images.) In other words, for each receptor/transporter, we calculate *N* choose 2 correlations (where *N* is the number of mean tracer images listed under “Other map(s)” in Table S1), then calculate their average. Note that out-of-sample mean receptor density maps may be collected using a different PET tracer. Furthermore, all MOR [$^{11}$C]carfentanil images were collected at the same PET centre and group maps may not be independent. Mean spatial consistency for MOR is therefore likely inflated.

### Coefficient of variation

In biological systems, the standard deviation of a distribution of measurements typically scales with the mean (Eisler et al. 2008) (see also Fig. S1 and Fig. S2). Therefore, rather than directly analyzing standard deviation values, we normalized the standard deviation by the mean. This ratio is called the coefficient of variation. In this work, we consider the coefficient of variation of tracer binding measurements (i.e. neurotransmitter receptor/transporter densities) both across individuals (“inter-indivudal”) and across regions (“inter-regional”). When calculated across individuals, there is one coefficient of variation value per region, representing inter-individual variability of within-region receptor/transporter density. The coefficient of variation can be unstable when the mean (denominator) approaches 0. Therefore, when calculating coefficient of variation, we omit the regions whose mean tracer binding is in the bottom fifth percentile, if tracer binding values are below 0.1.

Likewise, when calculated across regions rather than individuals, there is one inter-regional coefficient of variation value per brain map, representing how much receptor/transporter density varies across brain regions. More specifically, the standard deviation of mean tracer binding across brain regions (for cortex and subcortex separately) is divided by the mean tracer binding across brain regions. Finally, the regional-to-population coefficient of variation ratio is calculated as the inter-regional coefficient of variation divided by the mean inter-individual coefficient of variation. Values above 1 reflect neurotransmitter receptors/transporters that vary more across brain regions than across individuals, and values below 1 reflect neurotransmitter receptors/transporters that vary more across individuals than brain regions. We note that field-wide standards for “high” or “low” thresholds of coefficient of variability do not currently exist for PET tracer binding.

In Fig. 1 and Fig. S3 we test whether the inter-regional coefficient of variation is significantly greater than inter-individual coefficient of variation. Those receptors/transporters that show greater inter-regional than inter-individual coefficients of variation are more stably expressed across the brain and therefore group average normative maps are likely to be representative of the population. To conduct this statistical analysis, we bootstrap the distribution of inter-individual coefficient of variation (Fig. 3 and Fig. S3 histograms) 10 000 times and calculate the mean inter-individual coefficient of variation, resulting in a null distribution of 10 000 mean inter-individual coefficient of variations. We then compare the empirical inter-regional coefficient of variation with this null distribution ($p =$ number of times mean inter-individual coefficient of variation is greater than or equal to inter-regional coefficient of variation).

## Supplementary Information

Below is the link to the electronic supplementary material.

Supplementary file 1 (pdf 1015 KB)

## Data Availability

All code and data used to conduct the analyses are available at https://github.com/netneurolab/hansen_receptorvar.

## References

[CR1] Aghourian M, Legault-Denis C, Soucy J, Rosa-Neto P, Gauthier S, Kostikov A, Gravel P, Bedard M (2017) Quantification of brain cholinergic denervation in alzheimer’s disease using pet imaging with [18 f]-feobv. Mol Psychiatry 22(11):1531–1538 28894304 10.1038/mp.2017.183

[CR2] Alakurtti K, Aalto S, Johansson JJ, Någren K, Tuokkola T, Oikonen V, Laine M, Rinne JO (2011) Reproducibility of striatal and thalamic dopamine d2 receptor binding using [11c] raclopride with high-resolution positron emission tomography. J Cereb Blood Flow Metab 31(1):155–165 20442726 10.1038/jcbfm.2010.64 PMC3049480

[CR3] Alakurtti K, Johansson JJ, Joutsa J, Laine M, Bäckman L, Nyberg L, Rinne JO (2015) Long-term test-retest reliability of striatal and extrastriatal dopamine d2/3 receptor binding: study with [11c] raclopride and high-resolution pet. J Cereb Blood Flow Metab 35(7):1199–1205 25853904 10.1038/jcbfm.2015.53 PMC4640276

[CR4] Arumuham A, Nour MM, Veronese M, Onwordi EC, Rabiner EA, Howes OD (2023) The histamine system and cognitive function: An in vivo h3 receptor pet imaging study in healthy volunteers and patients with schizophrenia. J Psychopharmacol 37(10):1011–1022 37329185 10.1177/02698811231177287 PMC10612380

[CR5] Bäckman L, Nyberg L, Soveri A, Johansson J, Andersson M, Dahlin E, Neely AS, Virta J, Laine M, Rinne JO (2011) Effects of working-memory training on striatal dopamine release. Science 333(6043):718–718 21817043 10.1126/science.1204978

[CR6] Bäckman L, Waris O, Johansson J, Andersson M, Rinne JO, Alakurtti K, Soveri A, Laine M, Nyberg L (2017) Increased dopamine release after working-memory updating training: Neurochemical correlates of transfer. Sci Rep 7(1):7160 28769095 10.1038/s41598-017-07577-y PMC5540932

[CR7] Bedard M-A, Aghourian M, Legault-Denis C, Postuma RB, Soucy J-P, Gagnon J-F, Pelletier A, Montplaisir J (2019) Brain cholinergic alterations in idiopathic rem sleep behaviour disorder: a pet imaging study with 18f-feobv. Sleep Med 58:35–41 31078078 10.1016/j.sleep.2018.12.020

[CR8] Beliveau V, Ganz M, Feng L, Ozenne B, Højgaard L, Fisher PM, Svarer C, Greve DN, Knudsen GM (2017) A high-resolution in vivo atlas of the human brain’s serotonin system. J Neurosci 37(1):120–128 28053035 10.1523/JNEUROSCI.2830-16.2016 PMC5214625

[CR9] Bethlehem RA, Seidlitz J, White SR, Vogel JW, Anderson KM, Adamson C, Adler S, Alexopoulos GS, Anagnostou E, Areces-Gonzalez A et al (2022) Brain charts for the human lifespan. Nature 604(7906):525–533 35388223 10.1038/s41586-022-04554-y PMC9021021

[CR10] Buckner RL, Krienen FM (2013) The evolution of distributed association networks in the human brain. Trends Cogn Sci 17(12):648–665 24210963 10.1016/j.tics.2013.09.017

[CR11] Castrillon G, Epp S, Bose A, Fraticelli L, Hechler A, Belenya R, Ranft A, Yakushev I, Utz L, Sundar L et al. (2023) An energy costly architecture of neuromodulators for human brain evolution and cognition. Sci Adv 9(50):eadi7632 10.1126/sciadv.adi7632 PMC10848727 38091393

[CR12] Chang Z, McGinnity CJ, Hinz R, Wang M, Dunn J, Liu R, Yakubu M, Marsden P, Hammers A (2025) Machine learning to identify suitable boundaries for band-pass spectral analysis of dynamic [11 c] ro15-4513 pet scan and voxel-wise parametric map generation. EJNMMI Res 15(1):85 40643813 10.1186/s13550-025-01251-5 PMC12254443

[CR13] Cui Z, Li H, Xia CH, Larsen B, Adebimpe A, Baum GL, Cieslak M, Gur RE, Gur RC, Moore TM et al (2020) Individual variation in functional topography of association networks in youth. Neuron 106(2):340–353 32078800 10.1016/j.neuron.2020.01.029 PMC7182484

[CR14] Dagher A, Palomero-Gallagher N (2020) Mapping dopamine with positron emission tomography: a note of caution. Neuroimage 207:116203 31539589 10.1016/j.neuroimage.2019.116203

[CR15] DuBois JM, Rousset OG, Rowley J, Porras-Betancourt M, Reader AJ, Labbe A, Massarweh G, Soucy J-P, Rosa-Neto P, Kobayashi E (2016) Characterization of age/sex and the regional distribution of mglur5 availability in the healthy human brain measured by high-resolution [11 c] abp688 pet. Eur J Nucl Med Mol Imaging 43(1):152–162 26290423 10.1007/s00259-015-3167-6

[CR16] Eisler Z, Bartos I, Kertész J (2008) Fluctuation scaling in complex systems: Taylor’s law and beyond. Adv Phys 57(1):89–142

[CR17] Gallezot J-D, Nabulsi N, Neumeister A, Planeta-Wilson B, Williams WA, Singhal T, Kim S, Maguire RP, McCarthy T, Frost JJ et al (2010) Kinetic modeling of the serotonin 5-ht1b receptor radioligand [11c] p943 in humans. J Cereb Blood Flow Metab 30(1):196–210 19773803 10.1038/jcbfm.2009.195 PMC2949107

[CR18] Galovic M, Al-Diwani A, Vivekananda U, Torrealdea F, Erlandsson K, Fryer TD, Hong YT, Thomas BA, McGinnity CJ, Edmond E, et al. (2021a) In vivo nmda receptor function in people with nmda receptor antibody encephalitis. medRxiv

[CR19] Galovic M, Erlandsson K, Fryer TD, Hong YT, Manavaki R, Sari H, Chetcuti S, Thomas BA, Fisher M, Sephton S, et al. (2021b) Validation of a combined image derived input function and venous sampling approach for the quantification of [18f] ge-179 pet binding in the brain. NeuroImage: 118194 10.1016/j.neuroimage.2021.118194 34023451

[CR20] Hamati, R., Chidiac, B., Shvetz, C., Bdair, H., Dinelle, K., Holt, D. J., Cassidy, C., and Tuominen, L. (2025). Pavlovian fear conditioning, striatal dopamine release, and familial risk for psychosis. In preparation

[CR21] Hansen JY, Markello RD, Tuominen L, Nørgaard M, Kuzmin E, Palomero-Gallagher N, Dagher A, Misic B (2022) Correspondence between gene expression and neurotransmitter receptor and transporter density in the human brain. Neuroimage 264:119671 36209794 10.1016/j.neuroimage.2022.119671

[CR22] Hansen JY, Misic B (2025) Integrating and interpreting brain maps. Trends Neurosci 10.1016/j.tins.2025.06.003 40744776

[CR23] Hansen JY, Shafiei G, Markello RD, Smart K, Cox SM, Nørgaard M, Beliveau V, Wu Y, Gallezot J-D, Aumont É et al (2022) Mapping neurotransmitter systems to the structural and functional organization of the human neocortex. Nat Neurosci 25(11):1569–1581 36303070 10.1038/s41593-022-01186-3 PMC9630096

[CR24] Hansen JY, Shafiei G, Vogel JW, Smart K, Bearden CE, Hoogman M, Franke B, Van Rooij D, Buitelaar J, McDonald CR et al (2022) Local molecular and global connectomic contributions to cross-disorder cortical abnormalities. Nat Commun 13(1):1–17 35948562 10.1038/s41467-022-32420-y PMC9365855

[CR25] Huang W, Chen H, Liu Z, Dong X, Feng G, Liu G, Yang A, Zhang Z, Shmuel A, Su L, et al. (2025) Individual variability in the structural connectivity architecture of the human brain. J Neurosci 45(5) 10.1523/JNEUROSCI.2139-23.2024 PMC11780350 39667899

[CR26] Jaworska N, Cox SM, Tippler M, Castellanos-Ryan N, Benkelfat C, Parent S, Dagher A, Vitaro F, Boivin M, Pihl RO et al (2020) Extra-striatal d 2/3 receptor availability in youth at risk for addiction. Neuropsychopharmacology 45(9):1498–1505 32259831 10.1038/s41386-020-0662-7 PMC7360619

[CR27] Jiang Y, Palaniyappan L, Luo C, Chang X, Zhang J, Tang Y, Zhang T, Li C, Zhou E, Yu X et al. (2024) Neuroimaging epicenters as potential sites of onset of the neuroanatomical pathology in schizophrenia. Sci Adv 10(24):eadk6063 10.1126/sciadv.adk6063 PMC11168466 38865456

[CR28] Johansson J, Hirvonen J, Lovró Z, Ekblad L, Kaasinen V, Rajasilta O, Helin S, Tuisku J, Sirén S, Pennanen M et al (2019) Intranasal naloxone rapidly occupies brain mu-opioid receptors in human subjects. Neuropsychopharmacology 44(9):1667–1673 30867551 10.1038/s41386-019-0368-x PMC6785104

[CR29] Kaasinen V, Vahlberg T, Stoessl AJ, Strafella AP, Antonini A (2021) Dopamine receptors in parkinson’s disease: a meta-analysis of imaging studies. Mov Disord 36(8):1781–1791 33955044 10.1002/mds.28632

[CR30] Kantonen T, Karjalainen T, Isojärvi J, Nuutila P, Tuisku J, Rinne J, Hietala J, Kaasinen V, Kalliokoski K, Scheinin H et al (2020) Interindividual variability and lateralization of -opioid receptors in the human brain. Neuroimage 217:116922 32407992 10.1016/j.neuroimage.2020.116922

[CR31] Karahan E, Tait L, Si R, Özkan A, Szul MJ, Graham KS, Lawrence AD, Zhang J (2022) The interindividual variability of multimodal brain connectivity maintains spatial heterogeneity and relates to tissue microstructure. Commun Biol 5(1):1007 36151363 10.1038/s42003-022-03974-w PMC9508245

[CR32] Lamusuo S, Hirvonen J, Lindholm P, Martikainen I, Hagelberg N, Parkkola R, Taiminen T, Hietala J, Helin S, Virtanen A et al (2017) Neurotransmitters behind pain relief with transcranial magnetic stimulation-positron emission tomography evidence for release of endogenous opioids. Eur J Pain 21(9):1505–1515 28493519 10.1002/ejp.1052

[CR33] Laurikainen H, Tuominen L, Tikka M, Merisaari H, Armio R-L, Sormunen E, Borgan F, Veronese M, Howes O, Haaparanta-Solin M et al (2019) Sex difference in brain cb1 receptor availability in man. Neuroimage 184:834–842 30296558 10.1016/j.neuroimage.2018.10.013

[CR34] Luppi AI, Hansen JY, Adapa R, Carhart-Harris RL, Roseman L, Timmermann C, Golkowski D, Ranft A, Ilg R, Jordan D et al. (2023) In vivo mapping of pharmacologically induced functional reorganization onto the human brain’s neurotransmitter landscape. Sci Adv 9(24):eadf8332 10.1126/sciadv.adf8332 PMC10266734 37315149

[CR35] Majuri J, Joutsa J, Johansson J, Voon V, Alakurtti K, Parkkola R, Lahti T, Alho H, Hirvonen J, Arponen E et al (2017) Dopamine and opioid neurotransmission in behavioral addictions: a comparative pet study in pathological gambling and binge eating. Neuropsychopharmacology 42(5):1169–1177 27882998 10.1038/npp.2016.265 PMC5357051

[CR36] Majuri J, Joutsa J, Johansson J, Voon V, Parkkola R, Alho H, Arponen E, Kaasinen V (2017) Serotonin transporter density in binge eating disorder and pathological gambling: a pet study with [11c] madam. Eur Neuropsychopharmacol 27(12):1281–1288 29032922 10.1016/j.euroneuro.2017.09.007

[CR37] Markello RD, Hansen JY, Liu Z-Q, Bazinet V, Shafiei G, Suárez LE, Blostein N, Seidlitz J, Baillet S, Satterthwaite TD et al (2022) Neuromaps: structural and functional interpretation of brain maps. Nat Methods 19(11):1472–1479 36203018 10.1038/s41592-022-01625-w PMC9636018

[CR38] McGinnity CJ, Hammers A, Barros DAR, Luthra SK, Jones PA, Trigg W, Micallef C, Symms MR, Brooks DJ, Koepp MJ et al (2014) Initial evaluation of 18f-ge-179, a putative pet tracer for activated n-methyl d-aspartate receptors. J Nucl Med 55(3):423–430 24525206 10.2967/jnumed.113.130641

[CR39] McGinnity CJ, Riaño Barros DA, Hinz R, Myers JF, Yaakub SN, Thyssen C, Heckemann RA, Tisi JD, Duncan JS, Sander JW et al. (2021) Alpha 5 subunit-containing gabaa receptors in temporal lobe epilepsy with normal mri. Brain Commun 3(1):fcaa190 10.1093/braincomms/fcaa190 PMC7811756 33501420

[CR40] Monaghan A, Bethlehem RA, Akarca D, Margulies D, CALM T, Astle DE (2024) Canonical neurodevelopmental trajectories of structural and functional manifolds. bioRxiv: 2024–05 10.7554/eLife.103097 PMC13148822 42089485

[CR41] Morys F, Tremblay C, Rahayel S, Hansen JY, Dai A, Misic B, Dagher A (2024) Neural correlates of obesity across the lifespan. Commun Biol 7(1):656 38806652 10.1038/s42003-024-06361-9 PMC11133431

[CR42] Mosconi L, Nerattini M, Matthews DC, Jett S, Andy C, Williams S, Yepez CB, Zarate C, Carlton C, Fauci F et al (2024) In vivo brain estrogen receptor density by neuroendocrine aging and relationships with cognition and symptomatology. Sci Rep 14(1):12680 38902275 10.1038/s41598-024-62820-7 PMC11190148

[CR43] Mueller S, Wang D, Fox MD, Yeo BT, Sepulcre J, Sabuncu MR, Shafee R, Lu J, Liu H (2013) Individual variability in functional connectivity architecture of the human brain. Neuron 77(3):586–595 23395382 10.1016/j.neuron.2012.12.028 PMC3746075

[CR44] Murgaš M, Michenthaler P, Reed MB, Gryglewski G, Lanzenberger R (2022) Correlation of receptor density and mrna expression patterns in the human cerebral cortex. Neuroimage 256:119214 35452805 10.1016/j.neuroimage.2022.119214

[CR45] Nørgaard M, Beliveau V, Ganz M, Svarer C, Pinborg LH, Keller SH, Jensen PS, Greve DN, Knudsen GM (2021) A high-resolution in vivo atlas of the human brain’s benzodiazepine binding site of gabaa receptors. Neuroimage 232:117878 33610745 10.1016/j.neuroimage.2021.117878 PMC8256681

[CR46] Nørgaard M, Ganz M, Svarer C, Frokjaer VG, Greve DN, Strother SC, Knudsen GM (2019) Optimization of preprocessing strategies in positron emission tomography (pet) neuroimaging: a [11c] dasb pet study. Neuroimage 199:466–479 31158479 10.1016/j.neuroimage.2019.05.055 PMC6688914

[CR47] Nørgaard M, Ganz M, Svarer C, Frokjaer VG, Greve DN, Strother SC, Knudsen GM (2020) Different preprocessing strategies lead to different conclusions: a [11c] dasb-pet reproducibility study. J Cereb Blood Flow Metab 40(9):1902–1911 31575336 10.1177/0271678X19880450 PMC7446563

[CR48] Normandin MD, Zheng M-Q, Lin K-S, Mason NS, Lin S-F, Ropchan J, Labaree D, Henry S, Williams WA, Carson RE et al (2015) Imaging the cannabinoid cb1 receptor in humans with [11c] omar: assessment of kinetic analysis methods, test-retest reproducibility, and gender differences. J Cereb Blood Flow Metab 35(8):1313–1322 25833345 10.1038/jcbfm.2015.46 PMC4528005

[CR49] Palomero-Gallagher N, Zilles K (2019) Cortical layers: Cyto-, myelo-, receptor-and synaptic architecture in human cortical areas. Neuroimage 197:716–741 28811255 10.1016/j.neuroimage.2017.08.035

[CR50] Peckol EL, Troemel ER, Bargmann CI (2001) Sensory experience and sensory activity regulate chemosensory receptor gene expression in caenorhabditis elegans. Proc Natl Acad Sci 98(20):11032–11038 11572964 10.1073/pnas.191352498 PMC58678

[CR51] Pekkarinen L, Kantonen T, Oikonen V, Haaparanta-Solin M, Aarnio R, Dickens AM, Von Eyken A, Latva-Rasku A, Dadson P, Kirjavainen AK et al (2023) Lower abdominal adipose tissue cannabinoid type 1 receptor availability in young men with overweight. Obesity 31(7):1844–1858 37368516 10.1002/oby.23770

[CR52] Reardon P, Seidlitz J, Vandekar S, Liu S, Patel R, Park MTM, Alexander-Bloch A, Clasen LS, Blumenthal JD, Lalonde FM et al (2018) Normative brain size variation and brain shape diversity in humans. Science 360(6394):1222–1227 29853553 10.1126/science.aar2578 PMC7485526

[CR53] Ricard JA, Labache L, Segal A, Dhamala E, Cocuzza CV, Jones G, Yip SW, Chopra S, Holmes AJ (2024) A shared spatial topography links the functional connectome correlates of cocaine use disorder and dopamine d2/3 receptor densities. Commun Biol 7(1):1178 39300138 10.1038/s42003-024-06836-9 PMC11413242

[CR54] Rizzo G, Veronese M, Heckemann RA, Selvaraj S, Howes OD, Hammers A, Turkheimer FE, Bertoldo A (2014) The predictive power of brain mrna mappings for in vivo protein density: a positron emission tomography correlation study. J Cereb Blood Flow Metab 34(5):827–835 24496175 10.1038/jcbfm.2014.21 PMC4013760

[CR55] Saint-Georges Z, Shvetz C, Hamati R, Barara R, Dinelle K, Labelle A, Attwood D, Baines A, Owoeye O, Bdair H, Soucy J-P, Massarweh G, Solmi M, Cassidy C, Guimond S, Tuominen L (2025) Lower vesicular acetylcholine transporter in schizophrenia associates with cognitive deficits: an [18f]feobv pet study. In preparation

[CR56] Sandiego CM, Gallezot J-D, Lim K, Ropchan J, Lin S-F, Gao H, Morris ED, Cosgrove KP (2015) Reference region modeling approaches for amphetamine challenge studies with [11c] flb 457 and pet. J Cereb Blood Flow Metab 35(4):623–629 25564239 10.1038/jcbfm.2014.237 PMC4420880

[CR57] Savli M, Bauer A, Mitterhauser M, Ding Y-S, Hahn A, Kroll T, Neumeister A, Haeusler D, Ungersboeck J, Henry S et al (2012) Normative database of the serotonergic system in healthy subjects using multi-tracer pet. Neuroimage 63(1):447–459 22789740 10.1016/j.neuroimage.2012.07.001

[CR58] Schaefer A, Kong R, Gordon EM, Laumann TO, Zuo X-N, Holmes AJ, Eickhoff SB, Yeo BT (2018) Local-global parcellation of the human cerebral cortex from intrinsic functional connectivity mri. Cereb Cortex 28(9):3095–3114 28981612 10.1093/cercor/bhx179 PMC6095216

[CR59] Schoenberger M, Schroeder FA, Placzek MS, Carter RL, Rosen BR, Hooker JM, Sander CY (2018) In vivo [18f] ge-179 brain signal does not show nmda-specific modulation with drug challenges in rodents and nonhuman primates. ACS Chem Neurosci 9(2):298–305 29050469 10.1021/acschemneuro.7b00327 PMC5894869

[CR60] Segal A, Tiego J, Parkes L, Holmes AJ, Marquand AF, Fornito A (2025) Embracing variability in the search for biological mechanisms of psychiatric illness. Trends Cogn Sci 29(1):85–99 39510933 10.1016/j.tics.2024.09.010 PMC11742270

[CR61] Shafiei G, Fulcher BD, Voytek B, Satterthwaite TD, Baillet S, Misic B (2023) Neurophysiological signatures of cortical micro-architecture. Nat Commun 14(1):6000 37752115 10.1038/s41467-023-41689-6 PMC10522715

[CR62] Sieghart W, Sperk G (2002) Subunit composition, distribution and function of gaba-a receptor subtypes. Curr Top Med Chem 2(8):795–816 12171572 10.2174/1568026023393507

[CR63] Smart K, Cox SM, Scala SG, Tippler M, Jaworska N, Boivin M, Séguin JR, Benkelfat C, Leyton M (2019) Sex differences in [11 c] abp688 binding: a positron emission tomography study of mglu5 receptors. Eur J Nucl Med Mol Imaging 46(5):1179–1183 30627817 10.1007/s00259-018-4252-4 PMC6451701

[CR64] Smith CT, Crawford JL, Dang LC, Seaman KL, San Juan MD, Vijay A, Katz DT, Matuskey D, Cowan RL, Morris ED et al (2019) Partial-volume correction increases estimated dopamine d2-like receptor binding potential and reduces adult age differences. J Cereb Blood Flow Metab 39(5):822–833 29090626 10.1177/0271678X17737693 PMC6498753

[CR65] Sydnor VJ, Larsen B, Bassett DS, Alexander-Bloch A, Fair DA, Liston C, Mackey AP, Milham MP, Pines A, Roalf DR et al (2021) Neurodevelopment of the association cortices: patterns, mechanisms, and implications for psychopathology. Neuron 109(18):2820–2846 34270921 10.1016/j.neuron.2021.06.016 PMC8448958

[CR66] Talbot PS, Slifstein M, Hwang D-R, Huang Y, Scher E, Abi-Dargham A, Laruelle M (2012) Extended characterisation of the serotonin 2a (5-ht2a) receptor-selective pet radiotracer 11c-mdl100907 in humans: quantitative analysis, test-retest reproducibility, and vulnerability to endogenous 5-ht tone. Neuroimage 59(1):271–285 21782029 10.1016/j.neuroimage.2011.07.001 PMC3195881

[CR67] Tian Y, Margulies DS, Breakspear M, Zalesky A (2020) Topographic organization of the human subcortex unveiled with functional connectivity gradients. Nat Neurosci 23(11):1421–1432 32989295 10.1038/s41593-020-00711-6

[CR68] Tuominen L, Armio R-L, Hansen JY, Walta M, Koutsouleris N, Laurikainen H, Salokangas RK, Misic B, Hietala J (2025) Molecular, physiological and functional features underlying antipsychotic medication use related cortical thinning. Transl Psychiatry 15(1):129 40189580 10.1038/s41398-025-03336-0 PMC11973188

[CR69] Tuominen L, Nummenmaa L, Keltikangas-Järvinen L, Raitakari O, Hietala J (2014) Mapping neurotransmitter networks with pet: an example on serotonin and opioid systems. Hum Brain Mapp 35(5):1875–1884 23671038 10.1002/hbm.22298 PMC6869218

[CR70] Turtonen O, Saarinen A, Nummenmaa L, Tuominen L, Tikka M, Armio R-L, Hautamäki A, Laurikainen H, Raitakari O, Keltikangas-Järvinen L et al (2021) Adult attachment system links with brain mu opioid receptor availability in vivo. Biol Psychiat Cogn Neurosci Neuroimaging 6(3):360–369 10.1016/j.bpsc.2020.10.013 33431346

[CR71] Tyler WJ, Petzold GC, Pal SK, Murthy VN (2007) Experience-dependent modification of primary sensory synapses in the mammalian olfactory bulb. J Neurosci 27(35):9427–9438 17728456 10.1523/JNEUROSCI.0664-07.2007 PMC6673126

[CR72] Vernaleken I, Peters L, Raptis M, Lin R, Buchholz H-G, Zhou Y, Winz O, Rösch F, Bartenstein P, Wong DF et al (2011) The applicability of srtm in [18f] fallypride pet investigations: impact of scan durations. J Cereb Blood Flow Metab 31(9):1958–1966 21587267 10.1038/jcbfm.2011.73 PMC3185884

[CR73] Wiesman AI, Castanheira JDS, Fon EA, Baillet S, Group P-AR, Network QP (2024) Alterations of cortical structure and neurophysiology in parkinson’s disease are aligned with neurochemical systems. Ann Neurol 95(4):802–816 38146745 10.1002/ana.26871 PMC11023768

[CR74] Yang J, Chen K, Zhang J, Ma Y, Chen M, Shao H, Zhang X, Fan D, Wang Z, Sun Z et al (2023) Molecular mechanisms underlying human spatial cognitive ability revealed with neurotransmitter and transcriptomic mapping. Cereb Cortex 33(23):11320–11328 37804242 10.1093/cercor/bhad368

