# Supplementary material for: Inter-individual variability of neurotransmitter receptor and transporter density in the human brain
Source: Brain Struct Funct. 2026 Jan 13;231(1):13. doi: 10.1007/s00429-025-03069-2 (PMC12799681; doi:10.1007/s00429-025-03069-2)
Supplement: Supplementary file 1 — (pdf 1015 KB) [file 429_2025_3069_MOESM1_ESM.pdf]

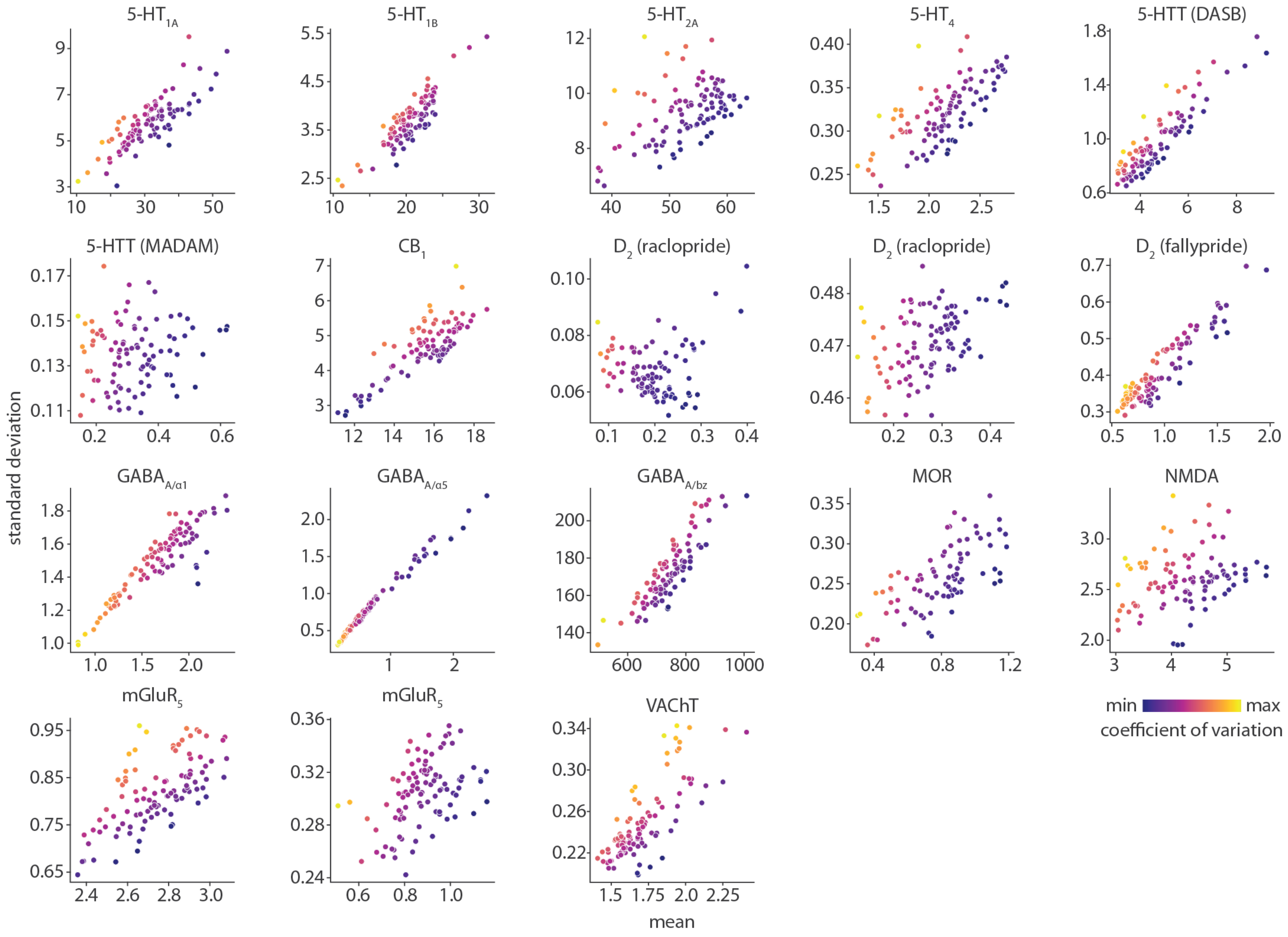

Figure S1. **Correlation between mean and standard deviation of receptor/transporter density within cortex** | Mean tracer binding (x-axis) is correlated with standard deviation of tracer binding (y-axis) across individuals. Each circle is a cortical region ($n = 100$). Circle colour represents inter-individual coefficient of variation (as shown in Fig. 1).

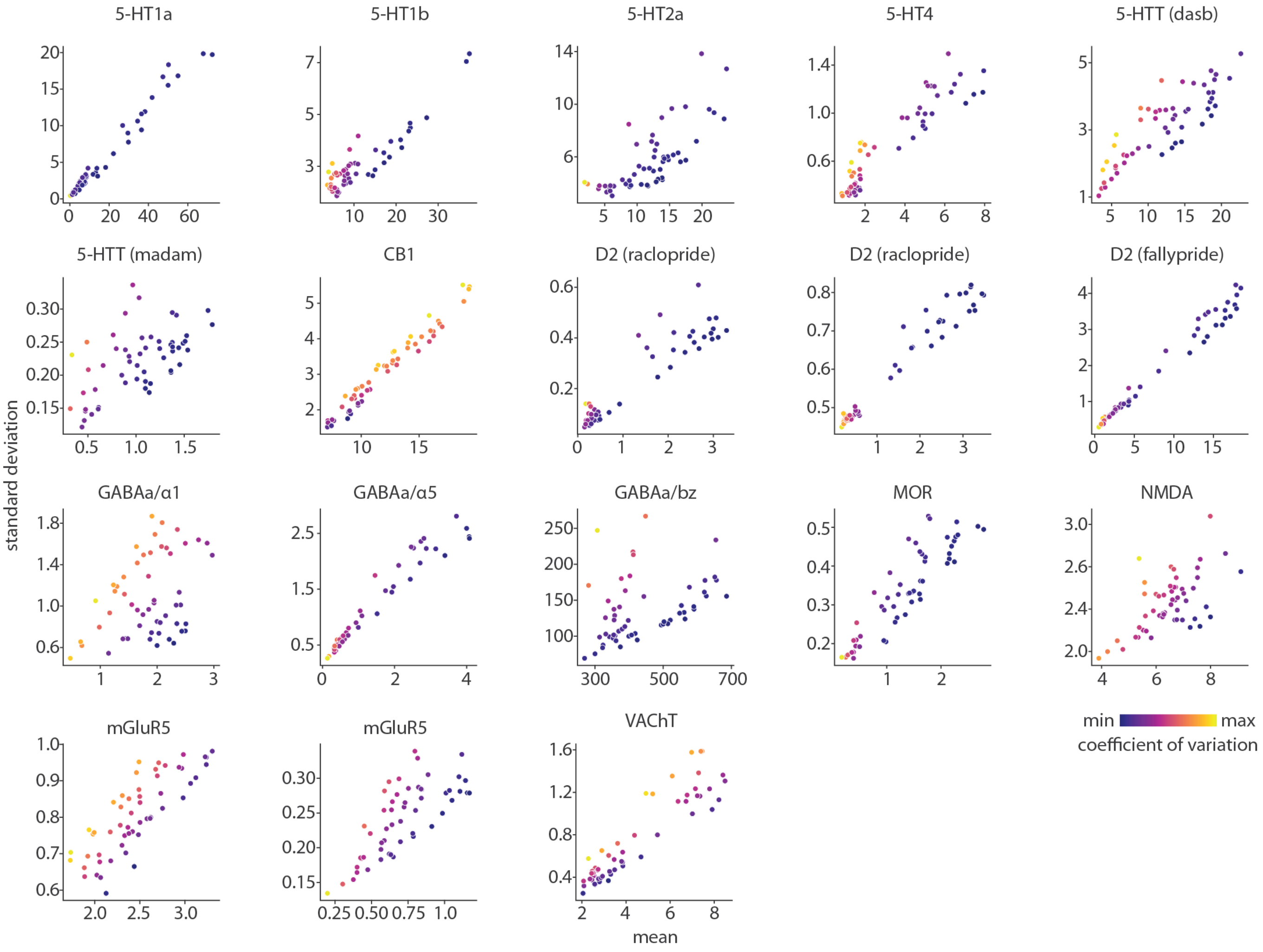

Figure S2. **Correlation between mean and standard deviation of receptor/transporter density within subcortex** | Mean tracer binding (x-axis) is correlated with standard deviation of tracer binding (y-axis) across individuals. Each circle is a subcortical region ($n = 54$). Circle colour represents inter-individual coefficient of variation (as shown in Fig. 2).

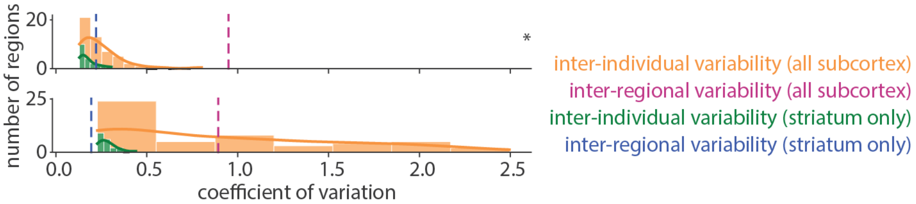

Figure S3. **Subcortical distributions of inter-individual coefficient of variation for D_2 [11C]raclopride tracer** | We show the distribution of subcortical inter-individual coefficient of variation (orange) and striatal inter-individual coefficient of variation (green). A kernel density is estimated for each distribution (solid lines). The y-axis represents the number of brain regions within each histogram bin, and the smooth curve represents the probability density estimate of the underlying histogram. The dashed purple line represents the inter-regional coefficient of variation across all subcortical structures, and the dashed blue line represents inter-regional coefficient of variation across all striatal regions. The asterisk in the top panel indicates that inter-regional variability across all subcortex is significantly greater than a null distribution of mean bootstrapped inter-individual coefficient of variation. Notably, variability is considerably lower in the striatum where [^{11}C]raclopride tracer is sensitive to D_2 receptor abundance. Data from [20] ($N = 16$) is shown on the top and data from [2, 5, 6] ($N = 47$) is shown on the bottom.

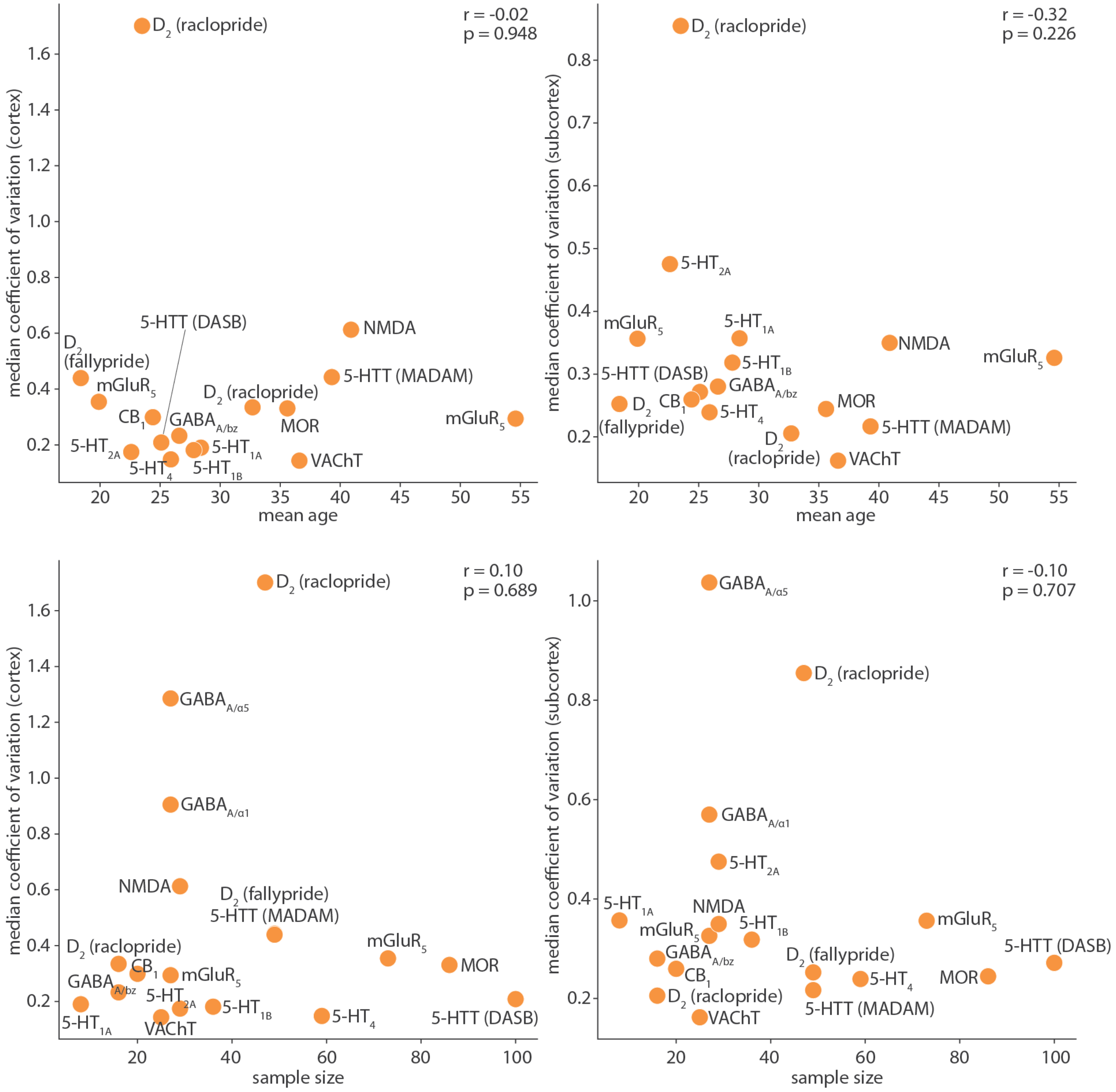

Figure S4. Median coefficient of variation (y-axis) of every receptor/transporter tracer for cortex (left) and subcortex (right) are correlated with mean sample age (top) and sample size (bottom).

Receptor/Transporter	Original map (N)	Other map(s) (N)
5-HT_{1A}	$[^{11}C]$CUMI-101 (8) [8]	$[^{11}C]$WAY-100635 (35) [57]
5-HT_{1B}	$[^{11}C]$AZ10419369 (36) [8]	$[^{11}C]$P943 (23) [57]<br>$[^{11}C]$P943 (65) [17]
5-HT_{2A}	$[^{11}C]$Cimbi-36 (29) [8]	$[^{18}F]$Altanserin (19) [57]<br>$[^{18}F]$MDL100907 (3) [66]
5-HTT	$[^{11}C]$DASB (100) [8]	$[^{11}C]$DASB (30) [57]<br>$[^{11}C]$MADAM (49) [36, 69]
5-HTT	$[^{11}C]$MADAM (49) [36, 69]	$[^{11}C]$DASB (30) [57]<br>$[^{11}C]$DASB (100) [8]
CB_1	$[^{18}F]$FMPEP-d_2 (20) [51]	$[^{18}F]$FMPEP-d_2 (22) [33]<br>$[^{11}C]$OMAR (77) [48]
D_2	$[^{11}C]$raclopride (16) [20]	$[^{11}C]$raclopride (47) [2, 5, 6]<br>$[^{11}C]$raclopride (7) [3]<br>$[^{18}F]$fallypride (49) [26]<br>$[^{18}F]$FLB457 (37) [64]<br>$[^{18}F]$FLB457 (55) [56]
D_2	$[^{11}C]$raclopride (47) [2, 5, 6]	$[^{11}C]$raclopride (16) [20]<br>$[^{11}C]$raclopride (7) [3]<br>$[^{18}F]$fallypride (49) [26]<br>$[^{18}F]$FLB457 (37) [64]<br>$[^{18}F]$FLB457 (55) [56]
D_2	$[^{18}F]$fallypride (49) [26]	$[^{18}F]$FLB457 (37) [64]<br>$[^{18}F]$FLB457 (55) [56]
$GABA_{A/\alpha 1}$	$[^{11}C]$Ro154513 (27; α_1) [39]	$[^{11}C]$Ro154513 (27; α_5) [39]<br>$[^{11}C]$flumazenil (16; BZ) [45]
$GABA_{A/\alpha 5}$	$[^{11}C]$Ro154513 (27; α_5) [39]	$[^{11}C]$Ro154513 (27; α_1) [39]<br>$[^{11}C]$flumazenil (16; BZ) [45]
$GABA_{A/BZ}$	$[^{11}C]$flumazenil (16; BZ) [45]	$[^{11}C]$Ro154513 (27; α_1) [39]<br>$[^{11}C]$Ro154513 (27; α_5) [39]
$mGluR_5$	$[^{11}C]$ABP688 (27) [15]	$[^{11}C]$ABP688 (73) [63]<br>$[^{11}C]$ABP688 (22) [23]
$mGluR_5$	$[^{11}C]$ABP688 (73) [63]	$[^{11}C]$ABP688 (27) [15]<br>$[^{11}C]$ABP688 (22) [23]
MOR	$[^{11}C]$carfentanil (86) [28, 32, 35, 69]	$[^{11}C]$carfentanil (204) [30]<br>$[^{11}C]$carfentanil (39) [70]
VAChT	$[^{18}F]$FEOBV (25) [55]	$[^{18}F]$FEOBV (5) [7]<br>$[^{18}F]$FEOBV (18) [1]

TABLE S1. **Out-of-sample group-average receptor/transporter density maps** | To calculate mean spatial consistency in Fig. 4, we correlate each receptor and transporter's mean tracer image ("original map") with any other available mean tracer image for this receptor/transporter ("other map(s)"), both from within the set of maps analyzed here, and from out-of-sample mean tracer images from the PET receptor atlas introduced in Hansen et al. [23]. Note that MOR $[^{11}C]$carfentanil maps were pulled from the same centre and therefore group maps are not necessarily independent.
